# Cystine reduces mitochondrial dysfunction in C2C12 myotubes under moderate oxidative stress induced by H_2_O_2_

**DOI:** 10.1007/s00726-022-03176-y

**Published:** 2022-06-18

**Authors:** Ami Mizugaki, Hiroyuki Kato, Tomoko Takeda, Yoshiko Inoue, Mai Hasumura, Tatsuya Hasegawa, Hitoshi Murakami

**Affiliations:** Institute of Food Sciences and Technologies, Ajinomoto Co., Inc, Kawasaki, Kanagawa Japan

**Keywords:** Cystine, Glutathione, Mitochondrial respiration, Muscle, Oxidative stress

## Abstract

Moderate oxidative stress induces temporal impairment in mitochondrial ATP production. As glutathione (GSH) content is reduced to eliminate oxidative stress by oxidation–reduction reaction, intracellular GSH content is crucial for maintaining mitochondrial function under oxidative stress. GSH precursors such as N-acetyl cysteine (NAC) and cysteine are known to suppress oxidative stress based on the supply of cysteine residues being rate-limiting for GSH synthesis. However, it remains unclear whether cystine (Cys2) can suppress mitochondrial dysfunction under oxidative stress conditions. Therefore, we examined whether Cys2 could attenuate mitochondrial dysfunction under moderate oxidative stress without scavenging reactive oxygen species (ROS) in the medium. C2C12 myotubes were incubated for 120 min in a Cys2-supplemented medium and subsequently exposed to hydrogen peroxide (H_2_O_2_). Heme oxygenase-1 (HO-1) gene expression, intracellular cysteine and GSH content, intracellular ATP level, and maximal mitochondrial respiration were assessed. Cys2 treatment significantly increased GSH content in a dose-dependent manner under oxidative stress. Cys2 treatment significantly decreased *HO-1* expression induced by H_2_O_2_ exposure. In addition, maximal mitochondrial respiration rate was decreased by H_2_O_2_ exposure, but improved by Cys2 treatment. In conclusion, Cys2 treatment mitigates oxidative stress-induced mitochondrial dysfunction by maintaining GSH content under moderate oxidative stress without scavenging ROS in the medium.

## Introduction

Oxidative stress is characterized by the overproduction of reactive oxygen species (ROS), primarily in the mitochondria (Brown and Borutaite 2012). Approximately 0.4–4.0% of all oxygen utilized by an organism is converted to superoxide in mitochondria, and then transformed to hydrogen peroxide (H_2_O_2_) by detoxification enzymes. However, the response to ROS varies depending on the severity and duration of exposure to ROS. High levels of ROS lead to irreversible mitochondrial damage, such as mitochondrial DNA mutations and apoptosis (Martindale and Holbrook 2002; Duan et al. 2005). In contrast, as H_2_O_2_ at moderate level reversibly oxidizes cysteine thiols (–SH) to sulfenic acid (-SOH), H_2_O_2_ temporally modifies several enzymes related to ATP production in mitochondria (Mailloux et al. 2014; Tretter and Adam-Vizi 1999; Hurd et al. 2008; Garcia et al. 2010). These modifications result in the impaired mitochondrial ATP production independent of cell death. The mitochondrial impairment is linked to a wide range of diseases (such as cardiovascular diseases, metabolic syndrome, insulin resistance, diabetes, and neurodegenerative diseases), and is the primary disease pathogenesis in some cases (Mailloux et al. 2014). Thus, these pathways may be targeted for therapeutic interventions.

Several anti-oxidative systems such as superoxide dismutase, glutathione peroxidases catalase, as well as nonenzymic compounds such as α-tocopherol (vitamin E), β-carotene, and ascorbate (vitamin C) have evolved to combat the accumulation of ROS (Martindale and Holbrook 2002; Fridovich 1995; Brigelius-Flohé and Maiorino 2013). Among these antioxidants, glutathione (GSH), one of the most abundant antioxidants in human cells, protects tissues against oxidative damage as well as maintains homeostasis and redox status (Schafer and Buettner 2001; Ookhtens and Kaplowitz 1998). The physiological role and metabolism of glutathione have been described in detail in previous review (Wu et al. 2004). Briefly, gamma glutamyl transpeptidase (GGT) plays key roles in GSH homeostasis by breaking down extracellular GSH and providing cysteine, which is the rate-limiting substrate for intracellular synthesis of GSH (Zhang et al. 2005). As oxidative stress induces the gene expression of GGT (Zhang et al. 2005), GSH precursors are required for synthesizing GSH as a substrate under oxidative stress conditions. Therefore, it is important to evaluate the role of GSH precursors in counteracting oxidative stress. N-acetyl-cysteine (NAC), a sulfhydryl group donor and GSH precursor (Sharma et al. 2016; Amini et al. 2016), protects mitochondrial ATP production and counteracts oxidative stress (Aparicio-Trejo et al. 2019; Lee et al. 2013). However, as some of GSH precursors such as NAC and cysteine have sulfhydryl groups that cause oxidation–reduction reactions with ROS sources in the medium, it has been proposed that the these GSH precursors directly scavenge ROS independent of glutathione synthesis (Zhang et al. 2011). Although previous studies have investigated the effects of GSH precursor pretreatment on anti-oxidative responses (Liu et al. 2019; Sharma et al. 2016; Lee et al. 2015), there are few studies investigating the effect of GSH precursors co-existing with ROS sources in the medium on anti-oxidative responses.

Cystine (Cys2) has a structure in which two cysteine molecules are connected via a disulfide bond (S–S), generated by the oxidation of the hydrosulfide group. Although cysteine mainly exists in the Cys2 form under normoxic conditions as it is rapidly oxidized to Cys2 in food, tissue-protein and blood, reducing condition make cysteine the prevailing form inside cells (Conrad and Sato 2012). Additionally, Cys2 is transported into muscle cells through the Cys2 transporter (xCT) that is expressed in muscle (Rebalka et al. 2019), and is converted into cysteine because intracellular status is in a reduced state. Cysteine is the rate-limiting precursor for GSH synthesis (Yin et al. 2016; Bannai and Tateishi 1986), but it is also metabolized to other compounds such as hypotaurine and taurine, which can react with free radicals. Thus, as Cys2 does not scavenge sources of oxidative stress, unlike NAC, thiazolidine, GSH, and cysteine, Cys2 can play a role of a GSH precursor under the condition with sources of oxidative stress, in the medium. However, the effect of Cys2 on mitochondrial dysfunction under oxidative stress has not yet been determined.

We aimed to clarify if Cys2, which is a GSH precursor that co-exists with oxidative stressors in the medium, can mitigate the mitochondrial dysfunction. We investigated the effect of Cys2 treatment in the presence of ROS sources in the medium, on mitochondrial oxygen consumption rate, GSH content, and gene expression of heme oxygenase-1 (HO-1), one of typical genes induced by oxidative stress.

## Materials and methods

### Reagents

Dulbecco’s Modified Eagle Medium (DMEM), Dulbecco’s phosphate buffered saline (DPBS), and penicillin–streptomycin-glutamine (100 ×) were obtained from Thermo Fisher (Waltham, MA, USA). Fetal bovine serum (FBS) was obtained from ICN Biomedicals (Costa Mesa, CA, USA). HEPES buffered saline solution was purchased from Lonza Ltd. (Walkersville, MD, USA). Trypsin–EDTA solution and L-cystine dihydrochloride were purchased from Sigma Chemical Company (St. Louis, MO, USA). Amino acid-free DMEM was purchased from Nacalai Tesque (Kyoto, Japan). Sodium hydrogen carbonate (NaHCO_3_), hydrogen peroxide (H_2_O_2_), 100 w/v% trichloroacetic acid solution (TCA), and 1 mol/L hydrochloric acid (HCl) were purchased from Wako Pure Chemical Industries (Osaka, Japan). Reduced glutathione (GSH) and dichloromethane were obtained from FUJIFILM Wako Pure Chemical Corporation (Osaka, Japan).

### Cell culture and oxidative stress with H_2_O_2_ exposure

**Fig. 1:**
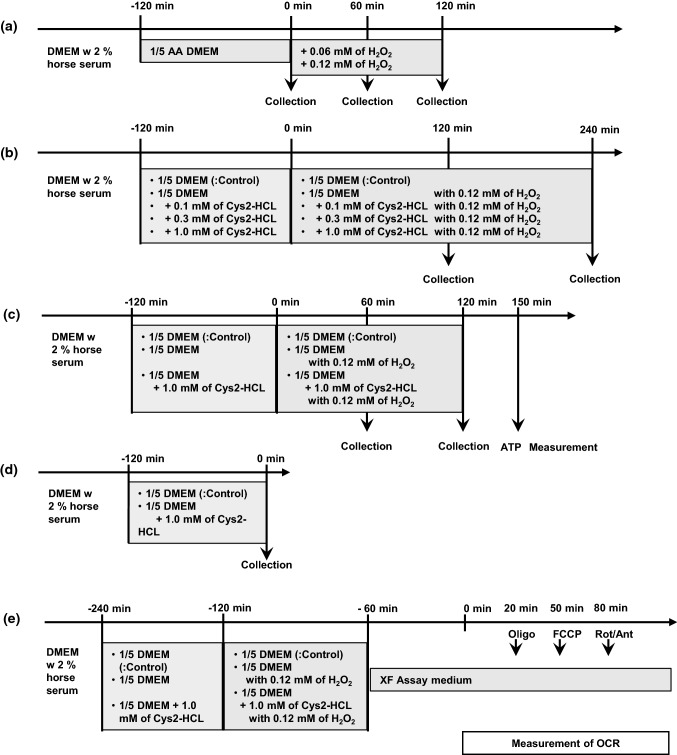
Experimental procedure. **a** To examine the effect of concentration of H_2_O_2_, C2C12 cells were incubated with 1/5 DMEM. After the incubation, the cells were exposed to 0.06 or 0.12 mM of H_2_O_2_.for 60 or 120 min. **b** To examine the effect of Cys2 on GSH content, cells were incubated with 1/5 DMEM medium or 1/5 DMEM supplemented with 0.1, 0.3 or, 1.0 mM Cys2-HCl for 120 min (b). After the incubation, the cells were exposed to 0.12 mM of H_2_O_2_ ± 1 mM Cys2-HCl for 120 or 240 min to test the effect of Cys2-HCL on GSH content in the cell. **c** to test the effect of 1 mM of Cys2 on cell viability, anti-oxidative reaction, the cells were exposed to 0.12 mM of H_2_O_2_ ± 1 mM Cys2-HCl for 120 min after 120 min of incubation with 1/5 DMEM ± 1 mM Cys2-HCl. **d**o test the 1 mM of Cys2 on GSH content before H_2_O_2_ exposure, the cells were collected after 120 min of incubation with 1/5 DMEM ± 1 mM Cys2-HCl. **e** A mitochondrial stress test was then performed to assess the bioenergetic status of the cells. Oxygen consumption rate (OCR) was recorded using the XFp Extracellular Flux Analyzers (Agilent Technologies, Santa Clara, CA). Cells were treated with the following chemicals at the respective time durations from the beginning of the assay: oligomycin at 20 min, carbonyl cyanide- 4-(trifluoromethoxy) phenylhydrazone (FCCP) at 50 min, and antimycin A and rotenone at 80 min. DMEM; Dulbecco’s Modified Eagle Medium; OCR, oxygen consumption ratio; Oligo, oligomycin (a complex V inhibitor); FCCP, carbonyl cyanide- 4-(trifluoromethoxy) phenylhydrazone; uncoupling agent); Rot/Ant, antimycin A and rotenone (a complex III and I inhibitor)

C2C12 myoblasts were purchased from KAC Co., Ltd. (Kyoto, Japan) and were grown at 37 °C and 5% CO_2_ in DMEM with 10% FBS, 1% penicillin–streptomycin-glutamine, and 1% HEPES. Myoblasts were seeded in 100-mm dishes at 1.8 × 10^4^ cells/mL/dish. C2C12 myoblasts at approximately 80–90% confluence were differentiated into myotubes upon further incubation in 2% horse serum for 2–3 days. The procedure of the experiments was summarized in Fig. 1. To examine the effect of content of H_2_O_2_, Differentiated C2C12 cells were incubated with 1/5 DMEM (DMEM medium diluted fivefold with amino acid-free DMEM). After the incubation, the cells were exposed to 0.06 or 0.12 mM of H_2_O_2_.for 60 or 120 min (Fig. 1a). To examine the effect of Cys2 on GSH content, cells were incubated with 1/5 DMEM medium or 1/5 DMEM supplemented with 0.1, 0.3 or, 1.0 mM Cys2-HCl for 120 min (Fig. 1b). As 1/5 DMEM medium contains 0.04 mM Cys2, final concentration of Cys2 in the medium were 0.14, 0.34 or 1.04 mM. After the incubation, the cells were exposed to 0.12 mM of H_2_O_2_ ± 1 mM Cys2-HCl for 120 or 240 min to test the effect of Cys2-HCL on GSH content in the cell (Fig. 1b). Following the exposure to Cys2-HCl, the cells were rinsed with DPBS. In previous studies, the effect of NAC on oxidative stress was examined with concentrations between the range of 1.0 mM to 5.0 mM (Zhang et al. 2011; Lee et al. 2015, 2013; Aparicio-Trejo et al. 2019). Therefore, we selected the highest concentration of Cys2-HCl for our experiment to be 1.0 mM, because this value was the lowest among the values of concentrations used in previous studies (Zhang et al. 2011; Lee et al. 2015, 2013; Aparicio-Trejo et al. 2019). In addition, we selected 0.1 and 0.3 mM of Cys2-HCL to test the effect of Cys2-HCL in physiological range. Then, we tested the effect of 1 mM of Cys2 on cell viability, anti-oxidative reaction (Fig. 1c). After 120 min of incubation with 1/5 DMEM ± 1 mM Cys2-HCl, the cells were exposed to 0.12 mM of H_2_O_2_ ± 1 mM Cys2-HCl for 120 min. In addition, to test the 1 mM of Cys2 on GSH content before H_2_O_2_ exposure, the cells were collected after 120 min of incubation with 1/5 DMEM ± 1 mM Cys2-HCl (Fig. 1d).

### Intracellular ATP measurements

Intracellular ATP was measured to assess cell viability (Maehara et al. 1987) using CellTiter-Glo™ (Promega Corporation, Madison, WI, USA) according to the manufacturer’s protocol. C2C12 myoblasts were seeded in 96-well plates and differentiated into myotubes. After 120 min of H_2_O_2_ exposure, the plate was equilibrated to room temperature for 30 min, and an equal volume (100 μL per well) of CellTiter-Glo™ reagent was added (Fig. 1c). Two minutes after mixing the contents of the plate, luminescence was detected using SpectraMax™ i3 (Molecular Devices Co., Sunnyvale, CA, USA).

### RNA extraction and real-time qPCR

Heme oxygenase-1 (HO-1) expression was measured as typical indicator of anti-oxidative response (Kurutas 2016). Total RNA was extracted according to the manufacturer's instructions (RNeasy Mini Kit ™, Qiagen, Hilden, Germany). RNA quantification and purity were estimated by spectrophotometry at 260 nm and 280 nm. Complementary DNA was synthesized using Prime Script™ RT reagent Kit (Takara, Shiga, Japan). HO-1 expression was quantified using SYBR Green (STBR® Green Real-Time PCR Master Mix, Thermo Fisher, Waltham, MA, USA) analysis with Quant Studio™ 12 K Flex Real-Time PCR System 384 well plate (Applied Biosystems, Inc., CA, USA). The following primers were used for HO-1: forward primer, 5'-TGCAGGTGATGCTGACAGAGG -3'; reverse primer, 5'- GGGATGAGCTAGTGCTGATCTGG-3′. The relative mRNA expression was calculated using cycle threshold (Ct) values and was normalized to the Ct values of 18S ribosomal RNA (18S rRNA) (forward primer, 5'-AACGCCACTTGTCCCTCTAA-3'; reverse primer, 5'-GTGGAGCGATTTGTCTGGTT -3').

### Measurement of cysteine, cystine, GSH and GSSG content

Cells were suspended in 50 μL of 10% trichloroacetic acid and mixed thoroughly following the addition of an equal amount of dichloromethane. The mixture was centrifuged at 14,000 × *g* for 2 min at 4 °C. Ten μL of the supernatant was diluted with 150 μL of 50 mM NaH_2_PO_4_, 1 mM 1-octanesulfonic acid sodium salt, and 2.5% (v/v) acetonitrile solution (pH 2.7). To test stability of cysteine, cystine, GSH and GSSG, the supernatant sample in the solution, the sample was analyzed after 24 h of storage at 4 degrees. As a results, the concentration was confirmed to be stable in the solution after deproteination for 24 h. Twenty μL of the sample was then separated using an Inertsil ODS-3 (5.0 μm, 3 × 150 mm column; GL Sciences Inc., Tokyo, Japan) with an HPLC system (GL-7410 pump, GL-7420 autosampler, GL-7430 oven, ED703 pulse electrochemical detector; GL Sciences Inc., Tokyo, Japan). The separation condition was previously described (Appala et al. 2016). A total of 50 mM NaH_2_PO_4_, 1 mM 1-octanesulfonic acid sodium salt, and 2.5% (v/v) acetonitrile solution (pH 2.7) were used as the mobile phase. The pump flow rate was set at 0.2 mL/min for 10 min and at 0.8 mL/min from 10 to 30 min after injection. Finally, the flow rate was maintained at 0.2 mL/min from 30 to 40 min after injection. The column oven temperature was maintained at 30 °C. The potential in the electrochemical detector was + 1800 mV. Retention time is 6.5 min for cystine, 7.5 min for cysteine, 11.3 for GSH, 20.3 for GSSG. The GSH concentration in the sample was calculated by comparing the peak area of the chromatogram with the calibration curve that was determined using a series of standard solutions with known concentrations of each substance. The concentration in the cells was calculated by multiplying the concentration of the analyzed sample by 16. The data below limit of detection (0.1 µM for cysteine, Cys2, GSH and GSSG) were expressed as N.D. As the samples had to be processed promptly when measuring glutathione-related metabolites, we did not measure necessary data (cell counts and cell weight etc.) for normalization. Therefore, the data were expressed the content per well.

### Mitochondrial oxygen consumption rate

A mitochondrial stress test was then performed to assess the bioenergetic status of the cells (Brand and Nicholls 2011) (Fig. 1e). C2C12 cells were differentiated into myotubes by incubation in XFp cell culture mini plates in a CO_2_ incubator at 37 °C. Following a 2 h treatment of Cys2, the C2C12 myotubes were exposed to H_2_O_2_ for 60 min. Before the assay, the cells were washed twice with XF Assay medium (Seahorse Bioscience, North Billerica, MA) containing 4.5 g/L glucose, 1.0 mM sodium pyruvate, and 4.0 mM glutamine (adjusted pH to 7.35 ± 0.05), and were incubated in a CO_2_-free incubator at 37 °C for 60 min. Following incubation, the baseline measurement of the oxygen consumption rate (OCR) was recorded as indicator of basal oxidative metabolism, using the XFp Extracellular Flux Analyzers (Agilent Technologies, Santa Clara, CA). Next, the cells were treated with the following chemicals at the respective time durations from the beginning of the assay: oligomycin (a complex V inhibitor, final concentration 3 μM) at 20 min to induce maximal glycolytic metabolism, carbonyl cyanide- 4-(trifluoromethoxy) phenylhydrazone (FCCP) (uncoupling agent, final concentration 3 μM) at 50 min to uncouple electron transport and induce peak OCR, and antimycin A and rotenone (a complex III and I inhibitor, respectively, final concentration 0.5 μM each) at 80 min to reveal non-mitochondrial respiration. Maximal respiration after the addition of FCCP reflects the maximal capacity of the electron transport chain (Ainscow and Brand 1999). Maximal respiration rate was calculated as the maximal OCR minus the non-mitochondrial OCR determined by antimycin A and rotenone treatment.

### Statistical analysis

All values are expressed as mean ± standard error. Comparison between the two groups was done using Sidak's multiple comparisons test followed by an analysis of variance test. For comparison among three groups, significant differences were determined using Tukey's multiple comparisons test or Dunnet’s multiple comparisons test. Data were analyzed using GraphPad Prism 7.04 software (GraphPad Software Inc., San Diego, CA, USA), with *p* < 0.05 being considered significant.

## Results

### Assay conditions and validation

**Fig. 2:**
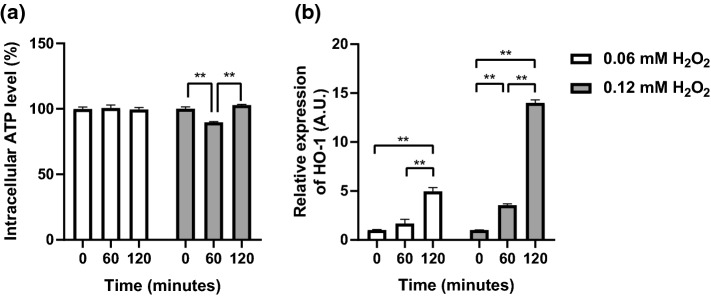
Time course changes of intracellular ATP level (**a**) and HO-1 gene expression (**b**) after H_2_O_2_ exposure in C2C12 skeletal muscle cells. Myotubes were incubated with 0.06 mM or 0.12 mM H_2_O_2_ for 60 or 120 min. Intracellular ATP levels w*n*as measured using CellTiter-Glo™ (Promega Corporation, Madison, WI, USA). HO-1 gene expression was measured by real-time qPCR. Data are shown as mean ± standard error (*n* = 3–12/group). * *p*< 0.05; **p < 0.01

No significant differences in intracellular ATP levels were observed over 120 min in 0.06 mM H_2_O_2_ (Fig. 2a). However, intracellular ATP level was significantly decreased at 60 min with 0.12 mM H_2_O_2_ concentration (*p* < 0.01), and significantly increased at 120 min with 0.12 mM H_2_O_2_ concentration, compared to that at 60 min (p < 0.01). However, no significant differences were observed between the ATP level at 0 min and 120 min with 0.12 mM H_2_O_2_ concentration (Fig. 2a)_._ Moreover, HO-1 gene expression, which is an oxidative stress marker, increased in a time-dependent manner with 0.06 mM and 0.12 mM H_2_O_2_ concentration (Fig. 2b, *p* < 0.01).

### Dose response of Cys2 on intracellular cysteine, cystine, GSH and GSSG contents

**Fig. 3:**
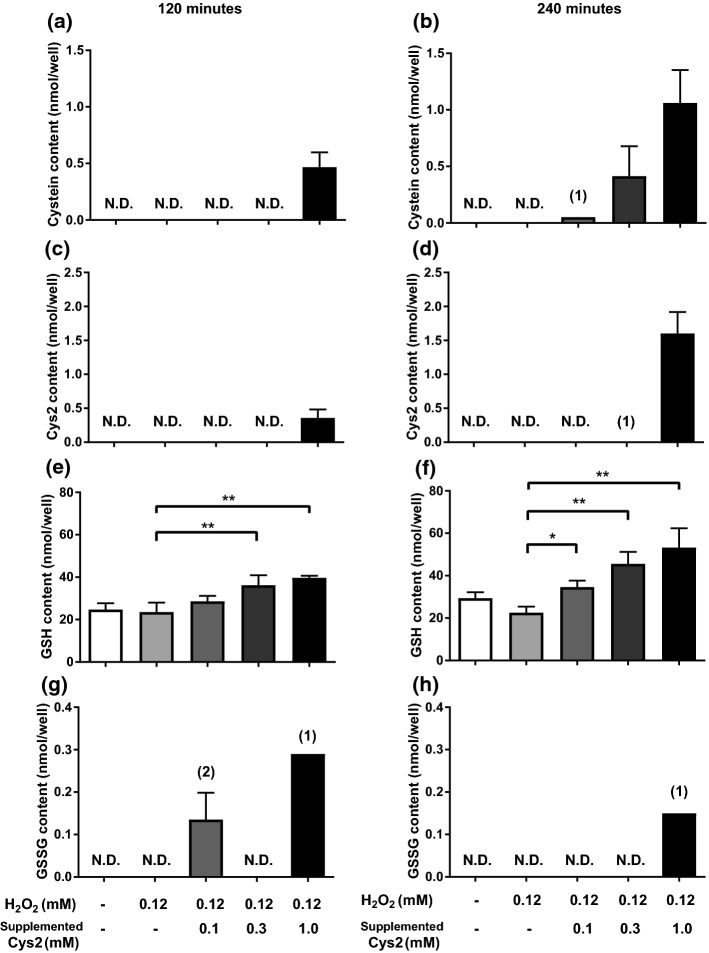
Effect of Cys2 on intracellular cysteine, cystine, GSH and GSSG contents after H_2_O_2_ treatment. Myotubes were incubated with 1/5 DMEM supplemented with 0, 0.1,0.3, or 1 mM Cys2 for 120 min, then exposed with 0.12 mM H_2_O_2_ for 120 (**a**, **c**, **e**, **g**) or 240 min (**b**, **d**, **f**, **h**). After cell was deproteinized with 10% TCA, GSH was extracted by dichloromethane. Cysteine (**a**, **b**), cystine (**c**, **d**), GSH (**e**, **f**) and GSSG (**g**, **h**) contents were measured by HPLC. Data are shown as mean ± standard error (*n* = 4/group). Parenthesis in figure shows n because some samples were not detected due to detection limit. *p < 0.05; **p < 0.01 (compared with 0.12 mM of H_2_O_2_)

After 120 min of Cys2 incubation, incubation with 0.3 mM or 1.0 mM of Cys2 increased intracellular cysteine and GSH content in dose-dependent manner, compared with H_2_O_2_ exposure without Cys2 (Fig. 3a, b, e, f). On the other hands, Cys2 and GSSG contents were shown in Fig. 3c, d, g, h. Because some samples were below limit of detection, sample number was decreased. Number of data was shown in parenthesis.

### Effect of Cys2 on intracellular ATP level and HO-1 gene expression

**Fig. 4:**
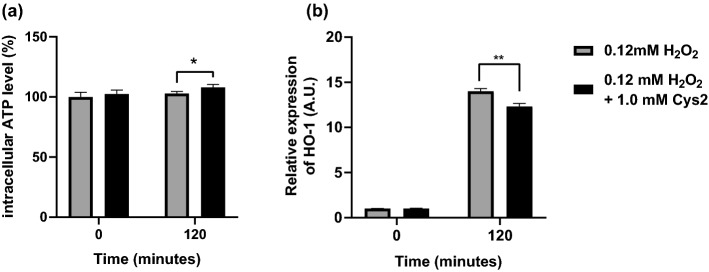
Effect of Cys2 on intracellular ATP level (**a**) and HO-1 gene expression (**b**) after H_2_O_2_ treatment Myotubes were incubated with or without 1 mM Cys2 for 60 min, then exposed with 0.06 mM H_2_O_2_ for 60 or 120 min. Intracellular ATP levels was measured using CellTiter-Glo™ (Promega Corporation, Madison, WI, USA). HO-1 gene expression was measured by real-time qPCR. Data are shown as mean ± standard error (*n* = 6/group). **p < 0.01

Intracellular ATP levels were not significantly different between the two groups before H_2_O_2_ treatment (*p* > 0.05, Fig. 4a). However, at 60 min and 120 min after H_2_O_2_ treatment (0.12 mM), the H_2_O_2_ + Cys2 group showed significantly increased intracellular ATP levels compared to those of the H_2_O_2_ group (p < 0.05, Fig. 4a). The HO-1 gene expression was also significantly suppressed in the H_2_O_2_ + Cys2 group compared to that of the H_2_O_2_ group at 120 min, but showed no significant suppression at 60 min (p < 0.01, p > 0.05, respectively, Fig. 4b).

### Effect of Cys2 without H_2_O_2_ exposure on intracellular cysteine, cystine, GSH and GSSG contents

**Fig. 5:**
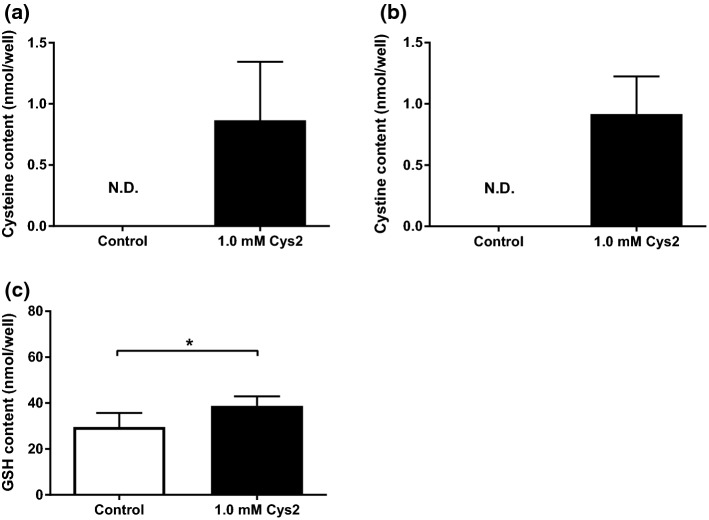
Effect of Cys2 without H_2_O_2_ exposure on intracellular cysteine (**a**), cystine (**b**) and GSH (**c**) contents. Myotubes were incubated with or without 1 mM Cys2 for 60 min, then collected. After cell was deproteinized with 10% TCA, GSH was extracted by dichloromethane. GSH content was measured by HPLC after extraction. Data are shown as mean ± standard error (*n* = 6/group). The data below detection limit was expressed as N.D. * *p*< 0.05

Cysteine and cystine contents were not detected in the control group, whereas detected in the Cys2 group (Fig. 5a, b). Cys2 incubation significantly increased GSH content at 120 min following treatment, compared to that of the control group (*p* < 0.05, Fig. 5c). GSSG was not shown because all the data were below limit of detection.

### Effect of Cys2 on mitochondrial respiration

**Fig. 6:**
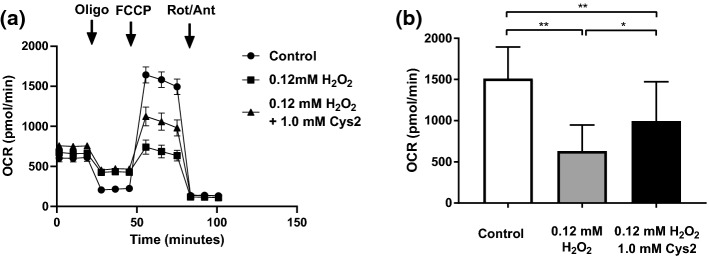
Effect of Cys2 on mitochondrial oxygen consumption after H_2_O_2_ treatment. Myotubes were incubated with or without 1 mM Cys2 for 60 min, then exposed with 0.06 mM H_2_O_2_ for 60 min. The medium was removed and replaced with the XF Assay medium 60 min prior the assessment of mitochondrial function. **a** Oxygen consumption ratio (OCR) was measured using the Extracellular Flux Analyzers XFp (Agilent Technologies, Santa Clara, CA) before and after sequential injections of the indicated compounds. Oligo, oligomycin (a complex V inhibitor, final concentration 3 μM) at 20 min; FCCP, carbonyl cyanide- 4-(trifluoromethoxy) phenylhydrazone; uncoupling agent, final concentration 3 μM) at 50 min; Rot/Ant, antimycin A and rotenone (a complex III and I inhibitor, respectively, final concentration 0.5 μM each). **b** Maximal respiration rate was calculated as maximal OCR minus non-mitochondrial OCR determined after antimycin A and rotenone. Data are shown as the mean ± standard error (*n* = 14–18/group). *p < 0.05; **p < 0.01. Con, incubation in 1/5 DMEM without H_2_O_2_ exposure

Figure 6a shows the change in OCR of the myotubes with addition of oligomycin, FCCP, rotenone/succinate, and antimycin A, respectively, as the experiment progressed. H_2_O_2_ treatment significantly decreased the mitochondrial maximal respiration rate compared to that of the control group that was incubated without H_2_O_2_ exposure (Con, *p* < 0.01, Fig. 6b), and the H_2_O_2_ + Cys2 group showed a significant suppression of the decline in mitochondrial maximal respiration rate compared to that of the H_2_O_2_ group (*p* < 0.05, Fig. 6b).

## Discussion

We examined the effect of Cys2 supplementation on GSH content, response to oxidative stress and mitochondrial oxygen consumption rate in C2C12 myotubes under H_2_O_2_ oxidative stress, to clarify if GSH precursor which co-exists with oxidative stressor in the medium, can mitigates the mitochondrial dysfunction without cell death. We found that Cys2 treatment reduces mitochondrial dysfunction of ATP production, increases GSH content and suppressed gene expression of anti-oxidative reaction enzyme (i.e. HO-1 expression).

In this study, we selected the moderate oxidative stress to induce mitochondrial dysfunction independent of cell death. Although oxidative stress-induced cell death irreversibly decreased intracellular ATP levels, the ATP level temporally decreased and then recovered in our study (Fig. 2a). Thus, cell death did not occur in the concentration (0.12 mM) of H_2_O_2_. Additionally, HO-1 gene expression, which is known to be induced by oxidative stress and to express the protein counteracting oxidative stress, significantly increased 14.0 times at 120 min compared with that at 0 min (pre-incubation) in 0.12 mM H_2_O_2_ (Fig. 2b). Furthermore, despite the concentration of H_2_O_2_ was relatively low compared with those in previous studies (Siu et al. 2009; Haramizu et al. 2017), mitochondrial maximal respiration was declined to the basal level by H_2_O_2_ treatment (Fig. 6a). In addition, ATP levels temporally decreased at 60 min after H_2_O_2_ treatment, but recovered at 120 min after H_2_O_2_ treatment (Fig. 2a). These results indicate that the moderate oxidative stress-induced mitochondrial dysfunction sufficiently and anti-oxidative stress response without cell death.

Under moderate oxidative stress, Cys2 which exists with H_2_O_2_ in the medium, increased GSH content (Fig. 3e, f) and decreased H_2_O_2_-induced increase in HO-1 gene expression (Fig. 4b). Furthermore, Cys2 supplementation increased GSH content without H_2_O_2_ stress (Fig. 5c). These results indicate that Cys2 treatment may decrease oxidative stress by maintaining GSH content. Although cysteine may be synthesized from methionine via the transsulfuration pathway in cells (McBean 2012), the supply of cysteine for the maintenance of intracellular GSH levels depends on extracellular Cys2 in cultured cells (Conrad and Sato 2012). Thus, these facts indicated that the extracellular Cys2 is utilized for maintaining GSH content. In our study, Cys2 supplementation increased intracellular cysteine content (Fig. 3a, b). xCT also plays a limiting role in the cellular supply of cysteine, which is the rate-limiting precursor to GSH (Yin et al. 2016; Bannai and Tateishi 1986), and in the biosynthesis of GSH (Dröge et al. 1994). Furthermore, as oxidative stress induces the expression of xCT and promotes GSH synthesis [21, 46], the extracellular Cys2 may be required to combat ROS in muscle cells under the oxidative stress condition.

Cys2 treatment improved mitochondrial maximal respiration evaluated as the OCR after the addition of the uncoupler FCCP (Fig. 6b). As FCCP is a potent uncoupler of mitochondrial oxidative phosphorylation and inflows protons into the inner membrane, the mitochondrial maximal respiration is assumed to reflect the mitochondrial respiratory chain complex activity. Furthermore, oxidative stress reduces mitochondrial respiratory capacity through decreasing mitochondrial respiratory chain complexes I (Aparicio-Trejo et al. 2019). Therefore, the decrease in mitochondrial maximal respiration means that the activity of the mitochondrial respiratory chain complex is directly decreased by H_2_O_2_ stimulation. As we mentioned above, Cys2 treatment decreased oxidative stress and increased GSH levels during H_2_O_2_ treatment. Thus, these results indicated that Cys2 reduced the decline in mitochondrial respiratory chain complexes induced by H_2_O_2_ treatment, through alleviating oxidative stress with maintaining GSH levels.

Surprisingly, intracellular ATP levels were increased by Cys2 treatment at 120 min after H_2_O_2_ treatment (Fig. 4a). This result indicates that Cys2 may enhance mitochondrial activity. NAC has been reported to stimulate protein synthesis in enterocytes, independently of glutathione synthesis (Yi et al. 2016). Thus, Cys2 may affect ATP levels independent of glutathione synthesis. However, GSH stimulates peroxisome proliferator-activated receptor-γ co-activator-1α (PGC-1α) protein expression in muscle (Aoi et al. 2015). PGC-1α is a key transcriptional co-activator, providing a mechanistic insight into nuclear regulatory pathways in mitochondrial biogenesis (Wu et al. 1999; Olesen et al. 2010; Finck and Kelly 2006) and controls gluconeogenesis and fatty acid oxidation (Gerhart-Hines et al. 2007). Thus, Cys2 may stimulate mitochondrial activity by increasing GSH synthesis and PGC-1α expression.

As Cys2 is the oxidized dimer form of cysteine and does not scavenge oxidative stressor in the medium, we supplemented Cys2 to determine if GSH precursor can mitigate the mitochondrial dysfunction and anti-oxidative response under oxidative stress. According to our current results, we speculated that extracellular Cys2 may be transported into the cell and increased intracellular cysteine content which is utilized for GSH synthesis. However, since we did not evaluate other cystine-metabolites such taurine, hypotaurine and so on, it remains unclear how Cys2 decreases oxidative stress. Thus, future studies might be required to determine extracellular Cys2 is utilized for GSH synthesis and/or the contribution of other Cys2-metabolites for decreasing oxidative stress.

A concentration of Cys2-HCl was selected as 0.1, 0.3 and 1.0 mM for our study. The highest concentration was 6.6-fold higher than the concentration of Cys2 in the basal 1 × DMEM (0.15 mM). In former study, oral administration of 200 mg/kg of Cys2 was shown to result in a threefold increase in plasma levels in mice (Kurihara et al. 2007). Therefore, we assumed that additional 0.1 and 0.3 mM of Cys2-HCL is within physiological level, while 1.0 mM of Cys2-HCL is barely the physiological level (or might be relatively high in the physiological levels). In our current study, we did not test the effect of low Cys2-HCL (i.e. 0.1 or 0.3 mM) on mitochondrial function. Therefore, validating the effect of cystine on mitochondrial function in vivo, and its physiological importance warrants future studies.

## Conclusion

We found that Cys2 co-existing with oxidative stressor in medium, decreased the oxidative stress-induced mitochondrial dysfunction independent of cell death. Cys2 supplementation also decreases oxidative stress response and increases intracellular GSH content. These results indicate that supplementation with Cys2 mitigates oxidative stress-induced mitochondrial dysfunction by maintaining intracellular GSH content.

## Data Availability

The datasets generated during and/or analyzed during the current study are available from the corresponding author on reasonable request.
